# Dual-Tuned Terahertz Absorption Device Based on Vanadium Dioxide Phase Transition Properties

**DOI:** 10.3390/ma17174287

**Published:** 2024-08-29

**Authors:** Ruyuan Zheng, Yingting Yi, Qianju Song, Zao Yi, Yougen Yi, Shubo Cheng, Jianguo Zhang, Chaojun Tang, Tangyou Sun, Qingdong Zeng

**Affiliations:** 1 Joint Laboratory for Extreme Conditions Matter Properties, Key Laboratory of Manufacturing Process Testing Technology of Ministry of Education, State Key Laboratory of Environment-Friendly Energy Materials, Southwest University of Science and Technology, Mianyang 621010, China; 2 College of Physics and Electronics, Central South University, Changsha 410083, China; 3 School of Physics and Optoelectronic Engineering, Yangtze University, Jingzhou 434023, China; shubocheng@yangtzeu.edu.cn; 4 School of Chemistry and Chemical Engineering, Jishou University, Jishou 416000, China; 5 Department of Physics, Jinzhong University, Jinzhong 030619, China; phys.zhangjg@gmail.com; 6 College of Physics, Zhejiang University of Technology, Hangzhou 310023, China; chaojuntang@126.com; 7 Guangxi Key Laboratory of Precision Navigation Technology and Application, Guilin University of Electronic Technology, Guilin 541004, China; 8 School of Physics and Electronic-Information Engineering, Hubei Engineering University, Xiaogan 432000, China

**Keywords:** terahertz, VO_2_, graphene, dynamic tunable

## Abstract

In recent years, absorbers related to metamaterials have been heavily investigated. In particular, VO_2_ materials have received focused attention, and a large number of researchers have aimed at multilayer structures. This paper presents a new concept of a three-layer simple structure with VO_2_ as the base, silicon dioxide as the dielectric layer, and graphene as the top layer. When VO_2_ is in the insulated state, the absorber is in the closed state, Δf = 1.18 THz (absorption greater than 0.9); when VO_2_ is in the metallic state, the absorber is open, Δf = 4.4 THz (absorption greater than 0.9), with ultra-broadband absorption. As a result of the absorption mode conversion, a phenomenon occurs with this absorber, with total transmission and total reflection occurring at 2.4 THz (A = 99.45% or 0.29%) and 6.5 THz (A = 90% or 0.24%) for different modes. Due to this absorption property, the absorber is able to achieve full-transmission and full-absorption transitions at specific frequencies. The device has great potential for applications in terahertz absorption, terahertz switching, and terahertz modulation.

## 1. Introduction

Terahertz waves are electromagnetic waves with frequencies in the range of 0.1–10 THz (wavelengths of 30–3000 μm), which overlap with millimetre waves in the long wavelength band and with infrared light in the short wavelength band [1]. The terahertz band is regarded as the gap between the microwave and infrared bands in the electromagnetic spectrum (hence the name “terahertz gap”), which has great research potential and application prospects [2]. In recent years, a large number of research works on terahertz imaging and terahertz wireless communication have been reported [3]. Terahertz technology is, of course, the technology that is seen as enabling 6G wireless technology, thus allowing the integration of the Internet of Things (IoT), covering a wide range of applications, and the realisation of fully intelligent and autonomous systems [4]. Besides, terahertz technology has great potential value in the military field. Therefore, an in-depth study of terahertz technology is necessary.

Graphene is an allotrope of carbon, the name given to a single layer of carbon atoms tightly packed in a two-dimensional (2D) honeycomb lattice, a single hexagonal honeycomb lattice structure formed by hybridisation of carbon atoms with sp^2^ [5]. Consisting of a single layer of tightly packed carbon atoms, the particle size is typically in the range of 10–100 nm, which represents good absorption in the mid-infrared and terahertz bands for graphene materials [6]. Graphene has excellent stability compared to other materials, excellent electrical conductivity and good hole electron mobility [7,8]. In addition to this, graphene has excellent mechanical and electromagnetic tunable properties and has become one of the most promising materials for tunable terahertz absorbing materials. The Fermi energy levels of graphene can be tuned by applying a bias voltage, thus achieving dynamically tunable absorption. Based on this excellent property, terahertz absorption in graphene-based structures has been extensively studied both theoretically and experimentally. Graphene materials have many advantages, but there are still many problems in their practical applications. But with the development of technology, its industrial applications are just around the corner [9].

Vanadium dioxide (VO_2_) is a typical PCM (phase change material) material with metal-insulator transition properties [10]. VO_2_ is considered a material for smart applications due to its metal-insulator phase transition (MIT) at 68 °C [11]. Due to its unique optical and infrared properties at different input phases, it has been widely used in the field of thermally controlled tunable. VO_2_ can transform between insulating and metallic states (i.e., undergo a phase transition) in response to external stimuli such as temperature, and the effect of temperature on its material properties is mainly reflected in changes in conductivity. During the heating process, when the temperature rises to around 340 K, the conductivity of VO_2_ increases sharply, and VO_2_ transforms from an insulating state to a metallic state [12]. During cooling, the conductivity of VO_2_ decreases dramatically when the temperature drops to about 330 K, and VO_2_ transforms from a metallic state to an insulating state. The conductivity can change by orders of magnitude (2 × 10^2^ S/m to 2 × 10^5^ S/m) during the transition between the insulating and metallic states, which provides ample possibilities for dynamic tuning of the devices [13].

Since the first proposal of absorbers by Landy et al. in 2008, absorber-related fields have been extensively studied [14]. In recent years, the proposed absorber structures based on new materials, such as graphene and VO_2_, have attracted wide attention. Compared with the absorbers processed by traditional materials, the new material absorbers have significant advantages in terms of tuning ability, absorption range, and absorption intensity [15,16,17]. Graphene-based absorbers can be tuned through the Fermi energy levels, thereby achieving enhanced absorber performance [18]. Absorbers based on VO_2_ can be characterised by their phase change properties, thereby producing ultra-broadband absorption [19]. As a result of research into terahertz technology, terahertz devices based on these novel materials have been heavily reported. Sun et al. proposed a dual-band independent tunable absorber, which consists of stacked graphene nanodiscs and graphene layers with nanopore structure and metal separated by an insulator layer, capable of producing two perfect absorption peaks [20]. Similarly, in 2023, a research group proposed a terahertz absorber composed of graphene and VO_2_ with dynamically variable double broadband absorption characteristics within the terahertz spectrum. In the metallic state of VO_2_, it exhibits more than 90% high-frequency absorption in the range of 8.65 THz to 10.30 THz; in the insulating state of VO_2_ at 0.7 eV, greater than 90% absorption occurs in the range of 2.85 THz to 4 THz [21].

In addition to this, multiple tunable absorbers based on hybrid structures of graphene and VO_2_ have been extensively studied, but due to the limitations of the current process, multilayer terahertz devices with similar multiple tuning are still some time away from achieving fabrication [22]. This paper presents a new concept of a three-layer simple structure with VO_2_ as the substrate, silicon dioxide as the dielectric layer, and graphene as the top layer. Graphene as the top layer ensures the stability of the absorber performance, while VO_2_ as the substrate extends the tunability of the absorber. The drastic changes in its conductivity and the unique metal–insulator transition of VO_2_ are well-suited for dynamic electromagnetic modulation materials [23]. When VO_2_ is in the insulated state, the absorber is off with absorber Δf = 1.18 THz (absorption greater than 0.9). When the VO_2_ is in the metallic state, the absorber is on, and there is ultra-broadband absorption with absorber Δf = 4.4 THz (absorption greater than 0.9). As a result of the absorption mode conversion, A = 99.45% (VO_2_ in the metallic state) and A = 0.29% (VO_2_ in the insulating state) at f = 2.4 THz. A = 90% (VO_2_ in the metallic state) and A = 0.24% (VO_2_ in the insulating state) at f = 6.5 THz. Due to this absorption property, the absorber is capable of switching between full-transmission and full-absorption at specific frequencies. Overall, the device has great potential for applications in terahertz absorption, terahertz switching, and terahertz modulation.

## 2. Modelling and Structural Parameters of the Absorber

**Figure 1:**
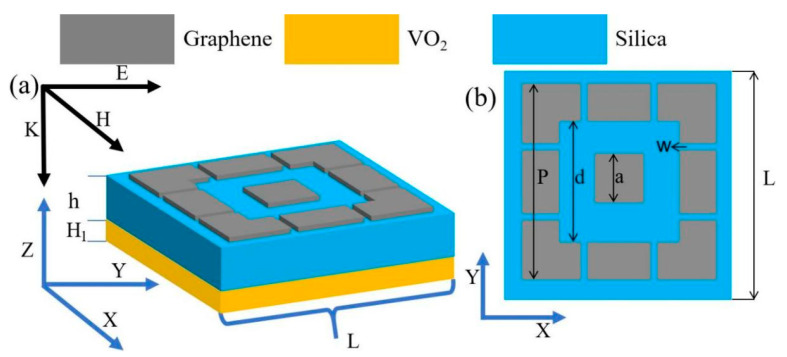
(**a**) Schematic diagram of the three-dimensional model; (**b**) schematic diagram of the plan structure.

The three-dimensional (3D) model of the dynamically tunable ultra-wideband absorber based on graphene metamaterial proposed in this paper is shown in Figure 1. Figure 1a shows the structural unit of the device, with a graphene-patterned layer at the top, a dielectric layer in the middle, and a VO_2_ substrate with a thickness of 0.5 µm at the bottom. The top graphene layer has a thickness of 1 nm, and the middle dielectric layer is a silica layer with a dielectric constant of ε=1.56. When VO_2_ is in the metallic state, its thickness is much greater than the skin depth of the incident electromagnetic wave, which ensures that the electromagnetic wave will not be transmitted [24]. Figure 1b shows the x–y plane schematic of the top graphene pattern, with a square with a side length in the centre, surrounded by four equal L-shaped patterns and four equal rectangles. The specific structural parameters are shown in Table 1.

**Figure 2:**
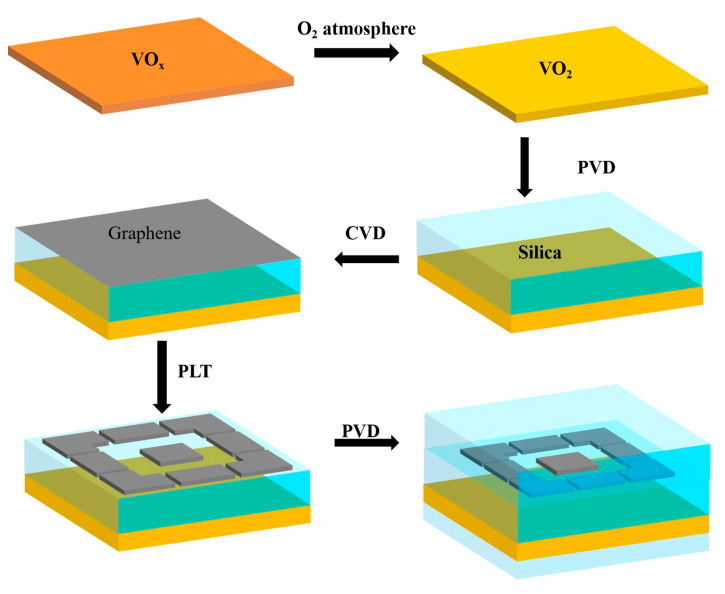
Schematic diagram of the manufacturing process.

Due to limited laboratory conditions, specific experiments could not be performed, but the methodology provided in this paper is feasible. Firstly, a 0.5 µm thick film of VO_x_ was deposited on a substrate containing a vanadium (V) metal target using a direct current magnetron sputtering (DC MS) method and further annealed in a low-pressure O_2_ atmosphere to convert the VO_x_ to VO_2_. Next, a silicon dioxide film with a thickness of 13 µm was deposited on a VO_2_ substrate using a physical vapour deposition (PVD) technique. Then, graphene is grown on the silica layer using the CVD technique and the desired pattern is obtained by photolithography [25]. Lastly, a silicon dioxide thin film was deposited on both sides of the device using the PVD method. A flow chart can be referred to below (Figure 2):

All the simulation results in this paper were obtained through CST Microwave Studio software. Periodic boundary conditions are set in the XY direction, and open boundary conditions are set in the z-direction; the electromagnetic wave propagates in the negative z-axis direction, and the mesh is adjusted to an adaptive mode to obtain higher accuracy [26]. In the terahertz frequency range, the derivation of the conductivity of monolayer graphene can be determined from the following equation based on Kubo’s formula [27]:

(1) $$\sigma \left(\omega \right)=\frac{e^{2}E_{f}}{\pi \hbar^{2}}\frac{i}{\omega +i\tau^{-1}}$$

ω denotes the incident wave frequency, E_f_ denotes the Fermi energy of graphene, ℏ denotes the approximate Planck constant, τ = $\mu E_{f}/ev_{f}^{2}$ denotes the relaxation time, μ = 10,000 cm^2^ V^−1^ s^−1^ denotes the carrier density, and the Fermi velocity V_f_ = c/300. The expression for its dielectric constant can be expressed as follows:

(2) $$\varepsilon =1+\frac{i\sigma \left(\omega \right)}{\varepsilon_{0}t\omega }$$

And for VO_2_, we can similarly use the Drude model to describe its dielectric constant in the terahertz range [28]:

(3) $${\varepsilon \left(\omega \right)}_{{VO}_{2}}=\varepsilon_{\infty }-\frac{\omega_{p2}^{2}}{\omega \left(\omega +i\gamma_{2}\right)}$$

$\varepsilon_{\infty }$ denotes the high-frequency relative permittivity of VO_2_, which can be set to a specific value of 12. γ denotes the collision frequency, with a value of 5.75 × 10^13^ rad/s, and ω is the frequency of the incident electromagnetic wave [29]. $\omega_{p}^{2}\left(\sigma \right)$ is the plasma frequency and the conductivity of VO_2_ satisfy the relationship equation [30]:

(4) $$\omega_{p}^{2}\left(\sigma \right)=\frac{\sigma }{\sigma_{0}}\omega_{p}^{2}\left(\sigma_{0}\right)$$

**Figure 3:**
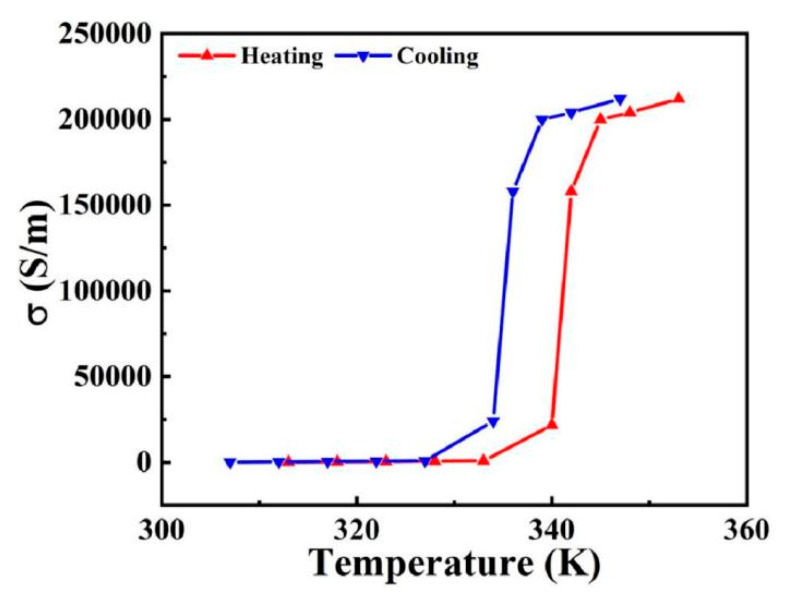
Conductivity versus temperature curves for VO_2_ films.

$\sigma_{0}$ = 3 × 10^5^ S/m, $\omega_{p}^{2}\left(\sigma_{0}\right)=1.4$ × 10^15^ rad/s. The operating conditions of VO_2_ at different temperatures can be realised by changing the conductivity in the corresponding simulation software [31]. When the temperature is changed, VO_2_ is able to achieve the transition between insulating and metallic states due to its unique phase transition properties, and the conductivity of VO_2_ versus ambient temperature is shown in Figure 3. Prior to the phase transition, VO_2_ is in an insulating state with a conductivity of 2 × 10^2^ S/m. When the temperature reaches the phase transition temperature, the VO_2_ is in a metallic state with a conductivity of 2 × 10^5^ S/m.

The S-parameter inversion method was used to determine the reflection coefficient R(ω) and transmission coefficient T(ω) of the device, whose absorption can be expressed as follows [32]:

(5) $$A(\omega )=1-R(\omega )-T(\omega )=1-{\left|S_{11}\right|}^{2}-{\left|S_{21}\right|}^{2}$$

ω is the angular frequency, R(ω) and T(ω) are the reflectance and transmittance, respectively, of the incident light wave when it irradiates the absorber unit structure, and S11 and S21 are the reflection coefficient and transmission coefficient, respectively. Reflectance is the percentage of total energy reflected from a substance, and transmittance is the ratio of the energy transmitted through a substance to the energy projected into it.

When VO_2_ is in the metallic state, the incident electromagnetic wave is completely reflected, and the transmittance T is negligible. Equation can be simplified as follows [33]:


(6) A = 1−R

The thickness of VO_2_ is much larger than its skin depth in the terahertz band, which can effectively isolate the transmittance phenomenon of the incident light wave, and it can be assumed that the transmittance T(ω) of this type of absorber is infinitely close to zero.

## 3. Results and Discussion

**Figure 4:**
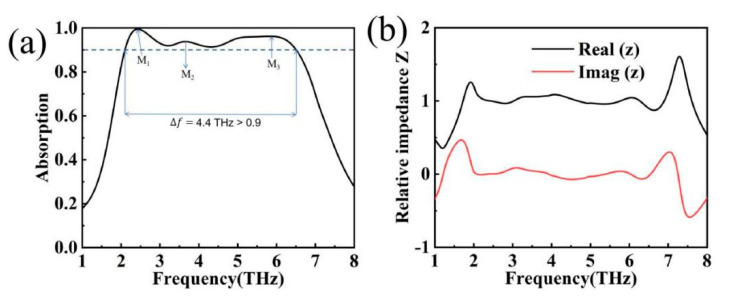
(**a**) Model absorption curve (M_1_ = 2.4 THz, M_2_ = 3.65 THz and M_3_ = 5.8 THz); (**b**) relative impedance plot.

As shown in Figure 4a, the absorber is capable of ultra-broadband absorption of 4.4 THz in the 2.1–6.5 THz interval (absorptivity greater than 0.9). The absorber has three main absorption points, M1, M2 and M3, with a perfect absorption peak at M1 and an absorption greater than 99%. In order to verify the accuracy of the simulation results, the relative impedance theory is introduced to invert the data, denoted as follows [34]:

(7) $$Z=\sqrt{\frac{{\left(1+S_{11}\right)}^{2}-S_{21}^{2}}{{\left(1-S_{11}\right)}^{2}-S_{21}^{2}}}$$

The impedance of the terahertz device matches perfectly with the free-space impedance when the real part of the impedance Re(Z) = 1 and the imaginary part of the impedance Im(Z) = 0, resulting in perfect absorption, as shown in Figure 4b. In the ultra-broadband absorption range (2.1 THz–6.5 THz), the direction of the impedance curve and the high absorption of the absorption map verify each other, with the real part of the impedance tending to 1 and the imaginary part of the impedance tending to 0 [35].

**Figure 5:**
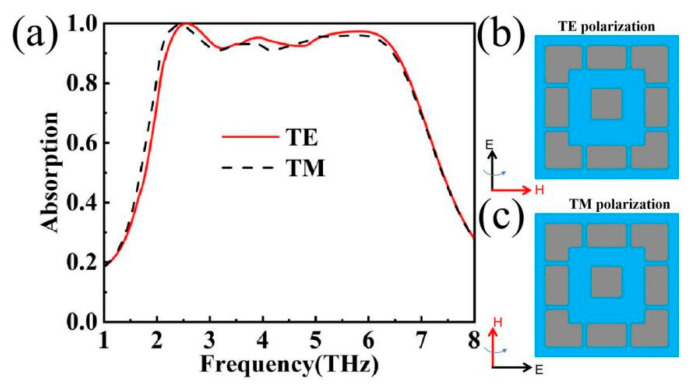
(**a**) Absorption maps in TE and TM modes; (**b**) TE-polarization schematic diagram; (**c**) TM-polarization schematic diagram.

In order to further explore the various parameters of the device, the variation of the absorptivity in two polarisation modes (TE and TM) was investigated, as shown in Figure 5a. Taking the x–y coordinate axis as a reference, the components in TE mode and TM mode are shown in Figure 5b,c. In the case of the structure proposed in this paper, similar absorption performance is maintained in both TE and TM modes, suggesting that there is great potential for the application of this device [36,37]. In the subsequent discussion of this paper, the discussion of each parameter focuses on the TE mode due to its similar absorption performance.

**Figure 6:**
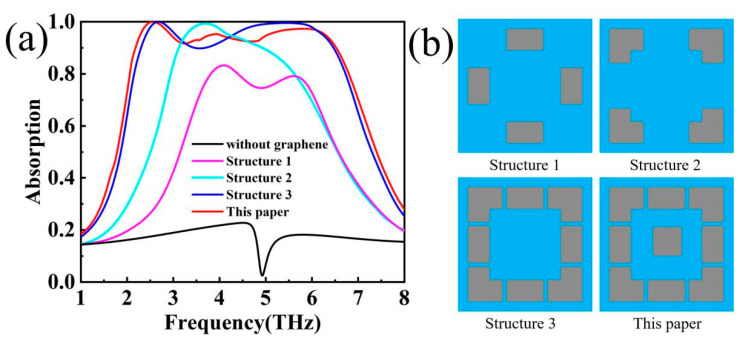
(**a**) Absorption diagram with different structures. (**b**) Schematic diagram of x–y plane with different structures.

In order to further verify the structural accuracy of this absorber, its decomposition combination is simulated, as shown in Figure 6. Metamaterial absorbers based on graphene structures have different combinations of surface structures and different coupling to electromagnetic waves [38]. Through the control variable method, the absorption under different structures of the device can be discussed in depth while keeping other physical quantities constant. As shown in Figure 6a, the Structure 1 absorption mode is at a low level of absorption, the Structure 2 absorption mode has a redshift in the low-frequency part, and the appearance of a perfect absorption peak at 3.5 THz, and the Structure 2 absorption mode has a high level of absorption. In the Structure 1 absorption mode, although there are two absorption peaks but the whole is at a low level of absorption, which may be due to the weak coupling between the device as a whole, and it is difficult to form a high absorption band with the electromagnetic wave [39]. In the Structure 2 absorption mode, there is a perfect absorption peak at 3.5 THz and high absorption in the nearly 1 THz band when the graphene layer has a strong interaction with the electromagnetic wave. The Structure 3 absorption mode has a high level of absorption due to strong interactions and is in perfect absorption at 5.2 THz–5.8 THz but is weak in the lower frequency band. Compared to Structure 3, the structure proposed in this paper loses the perfect absorption band of 5.2–5.8 terahertz but maintains a high level of absorption in the interval of 2.1 THz–6.5 THz (A > 0.9), which makes this choice worthwhile. Overall, there is essentially no absorption without the graphene structure, and the device jumps from no absorption and low-level absorption to high-level absorption through the combination of graphene structures. By studying the absorption curves under different structures, it can be shown that the structure proposed in this paper has the best absorption bandwidth.

**Figure 7:**
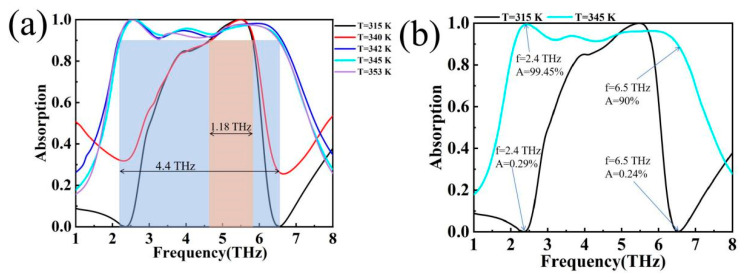
(**a**) Absorption curves corresponding to different temperatures for VO_2_ films, T = 315 K (σ = 2 × 10^2^ S/m), ∆*f* = 1.18 THz. T = 345 K (σ = 2 × 10^5^ S/m), ∆*f* = 4.4 THz (**b**) Absorption at T = 315 K and T = 345 K for the same frequency.

The terahertz device proposed in this paper has a strong tuning capability, which is mainly derived from the phase transition properties of VO_2_ material. When the VO_2_ substrate is in the insulating state, the device is at a low level of absorption (Δf = 1.18 THz); when the VO_2_ substrate is in the metallic state, the device has an ultra-broadband absorption (Δf = 4.4 THz), as shown in Figure 7a. This promising finding suggests that the selective nature of the device for electromagnetic wave absorption holds great promise in the dynamically tunable terahertz domain. For a more visual comparison, T = 315 K and T = 345 K are plotted in separate groups from Figure 7a. As shown in Figure 7b, when T = 315 K, VO_2_ is in an insulating state, the absorption rates corresponding to M1 and M3 are 0.29% and 0.24%, respectively, which can be approximated as the absorption rate A = 0. When T = 345 K, the VO_2_ is in the metallic state, and the absorption rates corresponding to M1 and M3 are 99.45% and 90%, respectively. Through the phase transition properties of the VO_2_ material, it is easy to see one of the magical properties of the device, which can be modulated by temperature to produce a response to a specific frequency [40]. In practical use, the device can be regulated by temperature, while in simulation software the effect of temperature can be investigated by the setting of conductivity parameters based on the relationship between temperature and conductivity [41].

**Figure 8:**
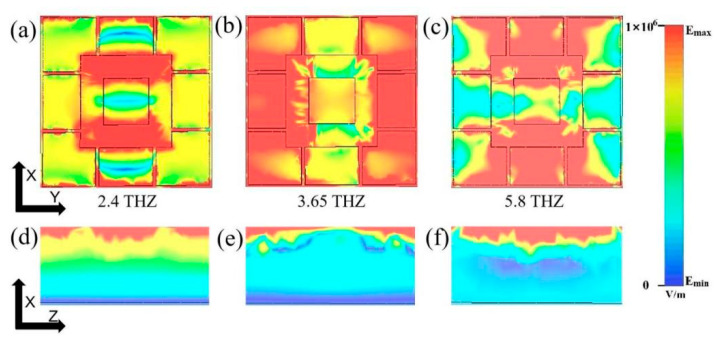
(**a**–**c**) represent the electric field in the xy-direction cross-section of the absorber at frequencies of 2.4 THz, 4.0 THz, and 5.8 THz, respectively; (**d**–**f**) represent the electric field on the xz-direction cross-section of the absorber for frequencies of 2.4 THz, 4.0 THz and 5.8 THz, respectively.

In order to further investigate the working mechanism of this terahertz device, it can be explored through its electric field diagram. Figure 8a–c shows the electric field absorption at f = 2.4 THz, f = 3.65 THz and 5.8 THz in the x–y plane, and Figure 8d–f show the electric field absorption in its x–z plane, respectively. The absorber proposed in this paper is similar to the MPA design, and the physical mechanism of the absorption response in this paper can be investigated according to the relevant principles [42]. The high absorption of the absorber is mainly caused by the displacement of the metamaterial resonance or the impedance-matching enhancement of the resonance unit to light. The strong absorption effect arises due to the electromagnetic wave interacting with the terahertz device structure and coupling with the structural modes to form a localised surface plasmon resonance (LSPR) [43,44]. In addition to this, VO_2_ is a metallic phase at high absorption, and metamaterials with conductive VO_2_ films form a typical MPA structure that behaves as a perfect absorber, as the structured top gold layer and the bottom continuous layer can now sustain both electric and magnetic resonance. The structure forms an ultra-broadband absorption in the presence of plasma resonance and electromagnetic resonance, forming a composite resonance [45,46]. It is possible to classify f = 2.4 THz, f = 3.65 THz and 5.8 THz as the low-frequency band, the middle-frequency band, as well as the high-frequency band of the absorption band and to define the portion of the graphene structure around the perimeter of the graphene structure as the edge module and the central square structure as the centre module. In the low-frequency band (f = 2.4 THz), the strong absorption region is concentrated at the centre position, with strong interactions between the L-type graphene in the edge modules and the centre module and between the gaps of each graphene module, resulting in high absorption. In the mid-frequency band (f = 3.65 THz), the strong absorption regions are concentrated in the edge modules on both sides of the y-axis of the device as well as between the gaps of the edge modules and the centre module, which may be due to the strong interactions of the graphene structure coupled with the electromagnetic waves in the mid-frequency band. In the high-frequency band (f = 5.8 THz), the strong absorption region is concentrated on both sides of the x-axis of the device and in the region of the graphene structure. Compared to the low-frequency and mid-frequency bands, the high-frequency band is weakly coupled at the edges, and the edge dipole resonance is weak.

**Figure 9:**
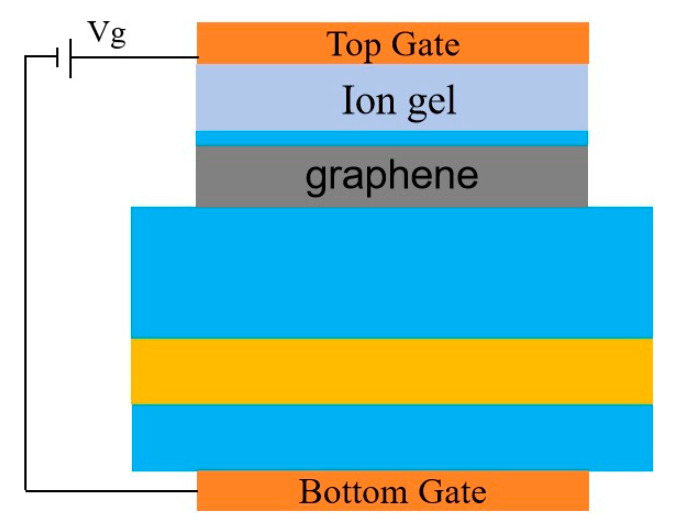
Schematic diagram of external bias voltage.

Graphene materials have great potential for optoelectronic functional applications, and the tunability of its Fermi energy level is a great advantage that other materials do not have. In this paper, the vanadium dioxide phase transition temperature is only 68 degrees Celsius, the excellent properties of graphene do not change, and the ionic gel is resistant to both high and low temperatures. Overall, the functionality of the device maintains excellent performance both before and after the vanadium dioxide phase transition. In addition, the respective modulation of the two is not affected by each other through two different modulation methods. The ion gel gate device used to tune the Fermi level of graphene is shown in Figure 9.

To achieve the modulation of the Fermi level in graphene, an ion gel was coated on the surface of the device, and an insulating layer was added at the bottom. To ensure the excellent performance of the device, ion gel materials with a refractive index close to that of the atmosphere can be used as the conductive medium. During the operation of the device, VO_2_ exists in the metallic state. Due to the skin effect of metals, the transmittance can be approximated as 0, and the influence of the insulating layer between the bottom connecting electrode and the VO_2_ layer can be neglected.

To further explore the tuning capability of the device, the Fermi level and relaxation time of graphene were investigated. The regulation of the graphene Fermi energy level can be adjusted by applying a bias voltage (*V_g_*), expressed as follows [47]:

(8) $$E_{f}=V_{f}\sqrt{\frac{\pi \varepsilon_{0}\varepsilon_{r}V_{g}}{e_{0}t_{d}}}$$

where td denotes the dielectric thickness, *V_g_* denotes the applied voltage, *V_f_* = c/300 is the Fermi velocity, $\varepsilon_{r}$ is the relative permittivity and $\varepsilon_{0}$ is the permittivity in a vacuum. According to this equation, the corresponding applied bias voltages *V_g_* = 1.73 V, 2.35 V, 3.07 V, 3.89 V and 4.8 V can be calculated for the set Fermi energy levels *E_f_* = 0.6 eV, 0.7 eV, 0.8 eV, 0.9 eV and 1.0 eV [48]. Since the structure proposed in this paper is a dispersed structure, an ionic gel can be used to coat the surface layer [49].

**Figure 10:**
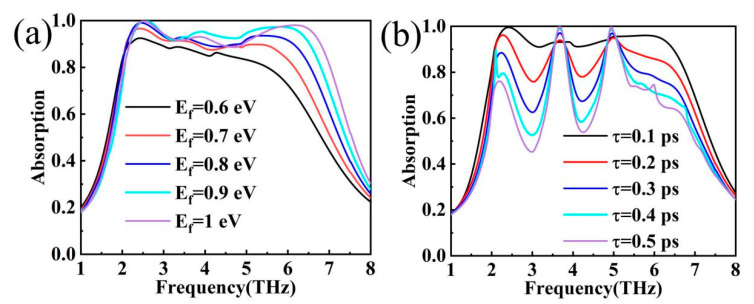
(**a**) Absorption curves of graphene at different Fermi energy levels; (**b**) absorption curves of graphene at different relaxation times.

As shown in Figure 10a, as the Fermi energy level rises from 0.6 eV to 1.0 eV, the absorption is gradually enhanced, and the high-frequency band undergoes a blueshift, which is converted from a low-level absorption to an ultra-broadband absorption. The position of the absorption peak at the main absorption point M1 in the low-frequency band remains essentially unchanged during the rise (*f* = 2.4 THz), and perfect absorption is achieved at *E_f_* = 0.8 eV. In addition, the relaxation time of graphene is also an important factor that affects the performance of the device. It is worth noting that the relaxation time of the material is determined at the completion of the preparation and cannot be changed. The device proposed in this paper has a relaxation time τ = 0.1 ps, and the subsequent study is only an exploratory experiment through simulation [50]. The relaxation time effect is mentioned here to increase the readability of the article and to provide a reference direction for subsequent related studies. As shown in Figure 10b, as the relaxation time of graphene increases, the ultra-broadband absorption is transformed into a narrow-band perfect absorption peak, which may be attributed to the increase in relaxation time, the rate of internal energy dissipation is accelerated, and the absorption intensity of the absorption peak is enhanced.

**Figure 11:**
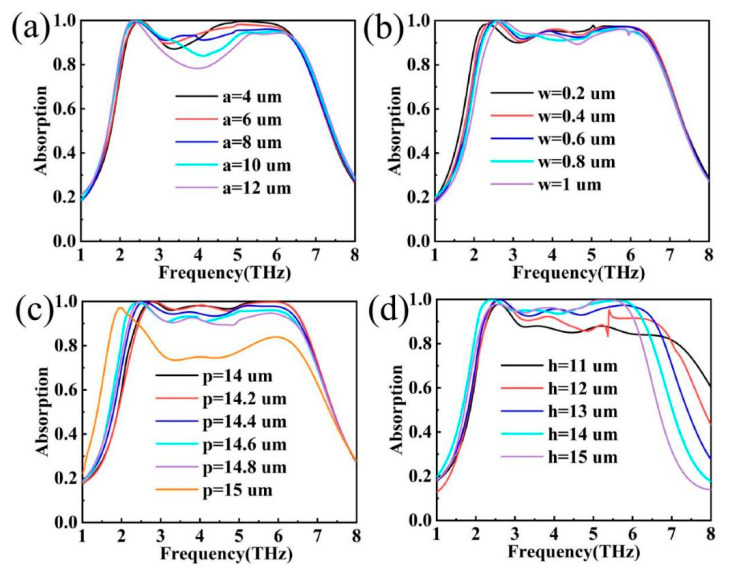
(**a**–**c**) Effect of structural parameters of graphene pattern on absorption; (**d**) effect of thickness h of dielectric layer silica on absorption.

In the actual manufacturing process, the structural parameters of the device itself are indispensable key factors, and due to the uncontrollable nature of the process, the error tolerance of the device itself is also an important criterion for judging the performance of the device [51,52]. In order to further explore the effect of structural parameters on the device, the physical parameters of the device can be explored by controlling the variables. All the parameters in this paper are optimal, and their specific values are detailed in Table 1. In practical applications, the effect of the physical parameters of the graphene structure on the absorption rate is shown in Figure 11a–c, and the effect of the thickness of the silica dielectric layer on the absorption rate is shown in Figure 11d. As shown in Figure 11a, the a-parameter represents the side length of the central square graphene structure, and the absorption profile is essentially unchanged when the a-parameter is varied in the range of less than 8 µm. When the a-parameter is larger than 8 µm, the absorption level decreases with increasing a-parameter, but the absorption peaks in the low-frequency band remain constant. As shown in Figure 11b, w denotes the slit width of the edge module, and the low-frequency band is blueshifted as the slit width increases, but the intensity of the low-frequency band absorption peak is almost constant. As shown in Figure 11c, the p-parameter represents the edge length of the whole graphene structure, and it is worth noting that the gradual increase of the p-parameter of the graphene structure from 14 µm to 15 µm shows a drastic change in the absorption profile at the increase of 14.8 µm to 15 µm, which may be due to the shift of the absorption mode of the device at p = 15 µm. The coupling between the structures of graphene is weakened, the resonance with electromagnetic waves is weakened, and the dipole resonance at the edges disappears [53,54]. As shown in Figure 11d, the absorption level increases, and the high-frequency band blueshifts as the h parameter of the silica dielectric increases. A comparison of the parameters reveals that size variations at the micrometre level around the optimum parameters have little effect on the performance of the device [55,56,57,58]. Overall, the device has some tolerance in fabrication tolerances and maintains its excellent performance up to a certain error.

**Figure 12:**
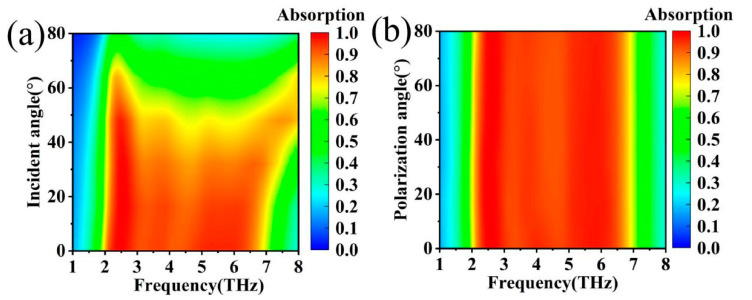
(**a**) Absorption spectra at different incidence angles; (**b**) absorption spectra at different polarisation angles.

In addition, in the process of practical application also need to consider the problem of electromagnetic wave incidence angle; different incidence angles of electromagnetic wave absorption spectrum may change [59,60,61,62]. As the angle of incidence increases, the high-frequency band in the absorption band is blueshifted, as shown in Figure 12a. Setting an interval of 10° over the angular variation of the incidence angle from 0° to 80°, a clear conclusion can be drawn that the absorption spectrum of the device remains unchanged over the range of incidence angles from 0° to 40°. It is worth noting that the absorption spectra of the terahertz device remain essentially unchanged when the polarisation angle of the incident wave is varied in the range of 0° to 80°, as shown in Figure 12b. To study the change in absorbance when the polarisation angle is varied, the range of the polarisation angle was similarly set to 0–80° with an interval of 10°. When the polarisation angle is varied in the range of 0–80°, the absorption spectrum is basically unchanged, as shown in Figure 12b. By studying the polarisation angle of the incident wave, an obvious conclusion can be drawn that the terahertz device proposed in this paper is insensitive to the polarisation angle [63,64,65,66]. VO_2_ and graphene materials are widely used in terahertz devices, and terahertz absorbers based on these two materials have been realised for functions such as narrow-band absorption and broadband absorption. The structure proposed in this paper offers superior performance compared to the various devices listed in Table 2 [67,68,69,70].

## 4. Conclusions

Overall, this paper innovatively presents a new concept of a three-layer simple structure with VO_2_ as the base, silicon dioxide as the dielectric layer, and graphene as the top layer, dexterously combining two emerging metamaterials. The graphene structure as the top layer ensures the stability of the absorber performance, and the VO_2_ substrate extends the dynamic tuning capability of the device. The transition from achieving full transmission and full absorption at specific frequencies and the expansion of ultra-wideband is achieved through sharp changes in conductivity. When VO_2_ is in the insulated state, the absorber is in the closed state absorber Δf = 1.18 THz (absorption > 0.9). When VO_2_ is in the metallic state, the absorber is switched on, and there is ultra-broadband absorption with absorber Δf = 4.4 THz (absorptivity > 0.9). In addition to this, the absorber is insensitive to changes in the angle of incidence from 0–40 degrees and insensitive to the angle of incidence polarisation from 0–80 degrees. Overall, due to the unique tuning characteristics, the device is able to achieve full-transmission and full-absorption transitions at specific frequencies, and the device has great potential for applications in terahertz absorption, terahertz switching, and terahertz modulation.

## Figures and Tables

**Table 1:** The specific structural parameters.

Parameter	a	d	L	w	p	h	H1
Value/μm	8	10	30	0.6	29.2	13	0.5

**Table 2:** Comparison of tunable ultrawideband absorbers.

Reference	Base	Metasurface	Relative Bandwidth (%) ^1^ Metallic State	Relative Bandwidth (%) ^1^ Insulated State	Number of Absorber Layers
[67]	VO_2_	VO_2_	82.5%	0	4
[68]	VO_2_/Copper	VO_2_/Copper	112%	0	6
[69]	VO_2_	Graphene	53%	0	3
[70]	Au	Graphene	54%	0	3
Proposed	VO_2_	Graphene	102%	22%	3

^1^ Bandwidth of absorption over 90%.

## Data Availability

Publicly available datasets were analysed in this study. These data can be found here: [https://www.lumerical.com/] (accessed on 1 January 2020).
